# Global timing: a conceptual framework to investigate the neural basis of rhythm perception in humans and non-human species

**DOI:** 10.3389/fpsyg.2014.00159

**Published:** 2014-03-03

**Authors:** Eveline Geiser, Kerry M. M. Walker, Daniel Bendor

**Affiliations:** ^1^Service de neuropsychologie et de neuroréhabilitation, Centre Hospitalier Universitaire VaudoisLausanne, Switzerland; ^2^Physiology, Anatomy, and Genetics, University of OxfordOxford, UK; ^3^Psychology and Language Sciences, Institute of Behavioural Neuroscience, University College LondonLondon, UK

**Keywords:** music, rhythm, grouping, meter, beat, tempo, brain, fMRI

## Abstract

Timing cues are an essential feature of music. To understand how the brain gives rise to our experience of music we must appreciate how acoustical temporal patterns are integrated over the range of several seconds in order to extract global timing. In music perception, global timing comprises three distinct but often interacting percepts: temporal grouping, beat, and tempo. What directions may we take to further elucidate where and how the global timing of music is processed in the brain? The present perspective addresses this question and describes our current understanding of the neural basis of global timing perception.

Rhythm perception is essential to our appreciation of music. Since music is a human construct and rhythm is defined primarily by its use in music, it is unclear whether studies of rhythm in non-human species directly relate to human rhythm perception. In this perspective, we use the term “global timing” as a conceptual framework, to emphasize the temporal computation required to perceive and recognize rhythm that is not unique to musical contexts, but is nonetheless required to perceive and recognize a musical rhythm. With this terminology, we also aim to facilitate the comparison of research findings across different animal models, where perception may take a different behavioral form but neural mechanisms may nevertheless be shared. We discuss the conceptual framework of global timing in light of recent research in both humans and other animal species, examine which brain regions may underlie global timing perception, and speculate on its potential neural mechanisms.

We define global timing as the percept of temporal patterns created by sequential acoustic events (i.e., notes and rests in a musical piece). It requires that the listener integrates temporal patterns spanning several seconds. While rhythm is also influenced by other acoustic characteristics such as pitch, intensity, and timber, global timing perception is, by definition, driven by timing. The human auditory system likely processes temporal patterns at two levels—it computes the *local* timing of individual events (e.g., the duration of a single note), and analyzes its *global* features; in human music perception, these include *temporal grouping*, *beat*, and *tempo* (Box 1A). Differences in the relative temporal proximity between musical notes can lead to temporal grouping perception, sometimes referred to as rhythmic or figural grouping. That is, musical notes that are relatively close in time appear to be functionally linked, thus inducing perceptual groups. A behavioral measure of how temporal proximity induces perceptual groups is the discrimination of grouping patterns across different tempi (Trehub and Thorpe, 1989), or the subjective elongation of time intervals between temporal groups (Geiser and Gabrieli, 2013). The beat is the expected timing of regularly recurring, often hierarchically structured, events in a piece of Western music, to which humans often clap or dance (Drake et al., 2000a). In its simplest form, the acoustic basis for beat perception is a sequence of temporally regular occurring sounds that repeat an identical inter-event-interval. The tempo in a musical sequence refers to how fast or slow the beat occurs (see also Box 1B). In the absence of beat, the perceived tempo will reflect the average rate of auditory events. Details on how these three terms are used in music perception are provided in reviews by McAuley (2010), Deutsch (2013), and Fitch (2013), and theoretical approaches to temporal pattern perception in humans have been summarized by Grahn (2012).

Box 1A **The framework of global timing with the three global temporal features in human music perception.**

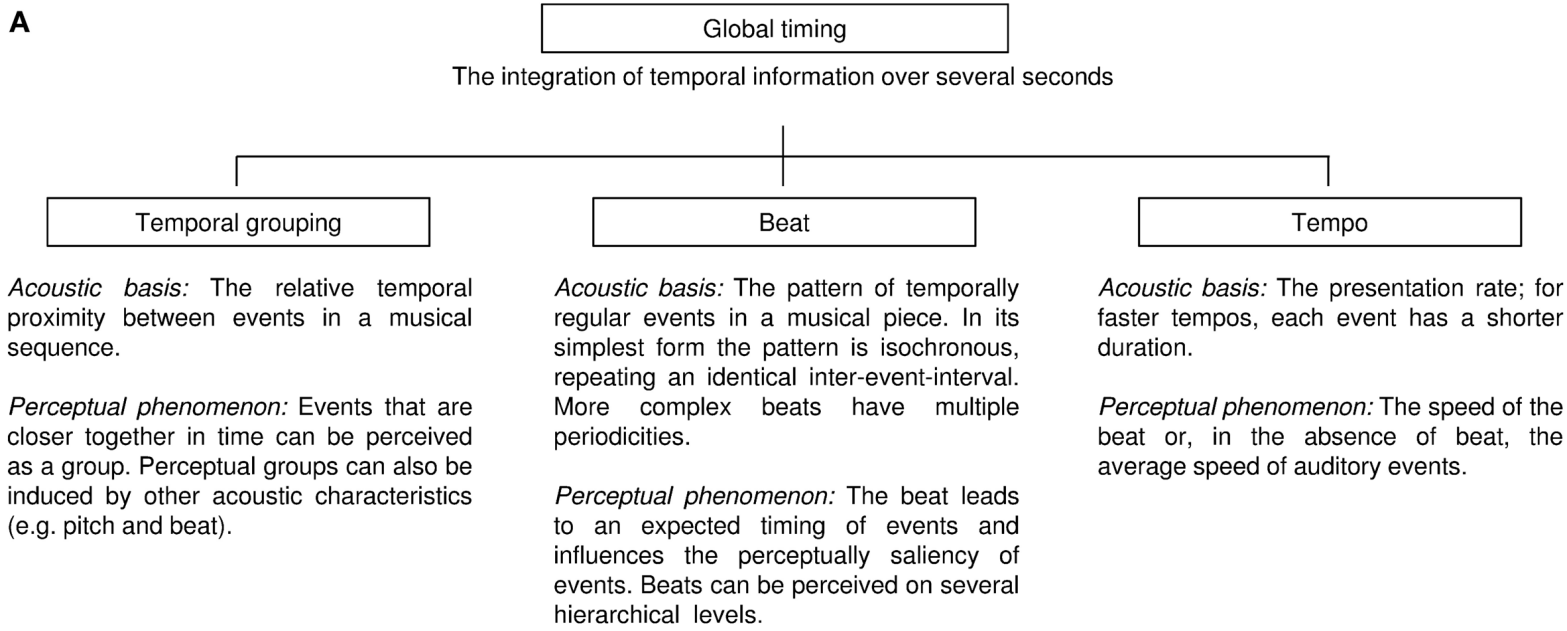

Box 1B **Musical notation of a temporal pattern in the French folk song “Sur le pont d'Avignon.”**

The schematic depiction indicates the relative tone onsets and duration on time axis (middle), of the perceptual groups based on temporal grouping (top), and the perceived beat (x) where number of stacked x's show the number of overlapping levels in the beat hierarchy (schema adapted from McAuley, 2010, p. 168, with kind permission from Springer Science and Business Media).

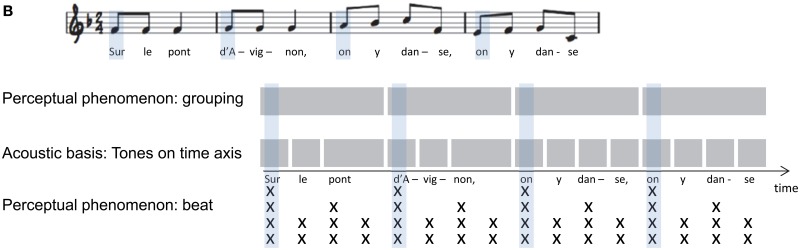

In music perception, beat, grouping, and tempo are *perceptually distinguishable*. This is, for example, reflected in their *perceptual invariance*. That is, a perceived temporal grouping pattern can be recognized across a range of different tempi (see Hulse et al., 1992 for a review). Also, the distinction between sequences comprising a beat and those comprising no beat, as well as the recognition of a tempo in two musical sequences, can be carried out across various grouping patterns. However, the perceptions of global timing features also influence each other. Temporal grouping can influence the perceived beat that, in turn, affects the perceived tempo (Povel and Essens, 1985). Moreover, the tempo can influence which level of beat is perceived in a musical sequence (Drake et al., 2000b) and beat perception can affect how musical notes are perceptually grouped (Povel and Essens, 1985; Clarke, 1987; Schulze, 1989; Desain and Honing, 2003; Fitch and Rosenfeld, 2007). Finally, there are individual differences in how these percepts interact (Grahn and McAuley, 2009). Age and musical experience can influence how we perceive global timing (Drake et al., 2000b; Geiser et al., 2010; Hannon et al., 2011, 2012). In general, humans tend to perceive temporally grouped tone sequences as multiples of a small symbolic duration, comprising an underlying temporal regularity or beat even when such underlying regularity is objectively lacking (Povel and Essens, 1985; Clarke, 1987; Schulze, 1989; Desain and Honing, 2003; ten Hoopen et al., 2006). Consequently, global timing in music comprises the percept of the three global temporal features as a conglomerate.

## Is global timing perception unique to humans?

Music is a human construct (for reviews on the evolutionary origin of music see McDermott and Hauser, 2005; Patel, 2006), but if non-human animals can be shown to share our perception of some features of global timing, then we can experimentally investigate the neural correlates of musical rhythm perception in these species. The characteristic temporal patterns of sounds produced by animals in nature (e.g., birdsong or the galloping of ungulates) suggest that many animals should be capable of global timing recognition. For example, the temporal pattern of vocal calls facilitates gender identification among emperor penguins (Jouventin et al., 1979) and species recognition in songbirds (Aubin and Brémond, 1983; Gentner and Hulse, 2000).

Can animals perceive specific features of global timing, such as temporal grouping, that are required for musical rhythm perception? Doves can detect a syllable delay within a conspecific vocalization (Slabbekoorn and ten Cate, 1999), which could reflect sensitivity to temporal grouping. Animals can also be trained to discriminate between non-ethologically relevant patterns for which temporal grouping might be a relevant cue (Ramus et al., 2000; Lomber and Malhotra, 2008). However, because many vocalizations also vary along non-temporal (e.g., spectral) dimensions, cues other than global timing could be used to differentiate these sounds. Further studies will be necessary to determine which species are able to distinguish grouping patterns solely on the basis of temporal cues.

The most striking difference in global timing perception between humans and many non-human species is that the latter exhibit a much poorer appreciation of beat. Human listeners readily detect and exploit temporal regularity in sound sequences. We remember auditory temporal patterns comprising a beat better than irregular patterns (Povel and Essens, 1985), and impose a regular beat on temporal patterns that we are required to remember (Povel, 1981). In contrast, animals have difficulty performing comparable tasks (pigeons: Hagmann and Cook, 2010; macaque monkeys: Zarco et al., 2009). Consequently, studies of beat perception in animals are rare. On the basis of our definition (Box 1A), one might expect that beat perception builds upon the basic ability to distinguish between temporally regular and irregular sound sequences. European starlings have been shown to discriminate between temporally regular (referred to as “rhythmic”) and irregular (referred to as “arrhythmic”) acoustic sequences and transferred the acquired categorization across different tempi (Hulse et al., 1984). However, attempts to train pigeons to categorize sound sequences in this manner have failed (Hagmann and Cook, 2010). When a temporally regular sequence of sounds was presented to Macaque monkeys, they did not show an electroencephalographic (EEG) response to beat deviants (Honing et al., 2012). This is in contrast to human newborns and adults, who show a mismatch-negativity response to beat deviants in similar stimuli (Winkler et al., 2009; Geiser et al., 2010). This finding suggests that not only do non-human primates fail to respond behaviorally to the beat, but they may also lack humans' neural correlates of beat perception.

Some parrots have been shown to synchronize their body movements to the beat of music due to model learning (Patel et al., 2009a,b) or even spontaneously (Schachner et al., 2009). Beat synchronization is one of the most commonly observed indicators for beat perception in humans from infancy onwards (Phillips-Silver and Trainor, 2005). The “vocal learning and rhythmic synchronization hypothesis” suggests that animals, such as parrots, who are vocal learners, might have the unique capacity to synchronize to the beat of sound sequences (Patel, 2006). However, beat synchronization has been recently observed in the California sea lion, a species presumed to be a non-vocal learner (Cook et al., 2013). While beat synchronization is likely a demonstration of beat perception, it does not need to be a requirement. The discrimination between sequences comprising temporal regularity and sequences comprising no regularity, as evidenced in the European starling (Hulse et al., 1984), suggests that beat perception might occur in species that lack beat synchronization. Whether this ability in starlings extends to more complex patterns found in global timing of music and can be found in other species remains to be investigated.

A number of species have been shown to discriminate the tempi of regular sound sequences, including birds (pigeons: Hagmann and Cook, 2010; European starlings: Hulse and Kline, 1993; emperor penguins: Jouventin et al., 1979; quails: Schneider and Lickliter, 2009), insects (crickets: Kostarakos and Hedwig, 2012), and non-human primates (cotton-top tamarins: McDermott and Hauser, 2007). Thus, it is likely that many animals share our perception of tempo. Moreover, like humans, starlings have been shown to ignore changes in tempo when categorizing the temporal pattern of sound sequences, relying instead on relative timing cues between acoustical events (Hulse and Kline, 1993). This perceptual invariance to tempo changes is a critical component of music perception in humans, as it allows us to recognize a familiar temporal pattern when it is played at a different speed. The finding that some animals share this aspect of global timing, at least when distinguishing regular and irregular sequences, is important as it means that we could study the neural mechanisms for tempo invariance at the neural level in these species.

In summary, we argue that various animals show sensitivity to the temporal grouping and the tempo feature of global timing in their spontaneous behavior and in experimental settings. Based on the above evidence one could speculate that some birds, such as European starlings, may also be able to distinguish the beat structure of sound sequences. The discussion of animal research above is selective, as we aimed to include only experimental paradigms that closely relate to perceptual mechanisms of global timing observed in humans.

## Which brain regions are implicated in global timing processing?

Functional Magnetic Resonance Imaging (fMRI) studies in healthy humans have associated a network of cortico-striatal brain areas [including the superior temporal gyrus (STG), the Supplementary Motor Area (SMA), prefrontal (PFC) and premotor cortices (PMC), the basal ganglia, and the cerebellum] with the performance of global timing tasks. These areas show increased activity compared to baseline when participants perform same-different judgments (Grahn and Brett, 2007; Chen et al., 2008), categorization (Geiser et al., 2008), and tempo judgments on temporal patterns (Henry et al., 2013), or when they passively listen to temporal patterns (Bengtsson et al., 2008). The global timing features investigated in these studies included beat and tempo. This network of brain areas is, however, not exclusively linked to global timing in the auditory domain. It is also implicated in local timing (for a review see Bueti, 2011). Specifically, activity in the cerebellum and the basal ganglia has been observed during duration discrimination, both in the auditory (Rao et al., 2001; Belin et al., 2002) and visual modality (Coull and Nobre, 1998; Ferrandez et al., 2003; Lewis and Miall, 2003; Coull, 2004; Pouthas et al., 2005). Taken together, these findings could indicate a general temporal processing function for these brain areas (Teki et al., 2012). Alternatively, these brain areas could differ functionally on the micro-anatomical level. Such functional distinctions could be found in direct comparisons of local and global timing tasks using high-resolution imaging.

There is evidence for a functional distinction between cortical and subcortical brain areas of the network identified in global timing perception studies. The putamen, which is part of the basal ganglia, is associated specifically with beat and temporal regularity perception. In a rhythm discrimination task, increased activity in the putamen was observed for temporal patterns comprising temporal regularity compared to rhythms comprising no regularity (Grahn and Brett, 2007). This association of the putamen with beat perception was replicated in passive listening (Bengtsson et al., 2008), temporal pattern reproduction (Chen et al., 2008), pitch and intensity change detection (Grahn and Rowe, 2009; Geiser et al., 2012), and duration perception tasks (Teki et al., 2011). This suggests that the putamen responds to temporal regularity in auditory stimuli and that this response even occurs when listeners do not perform a temporal task. Future studies will need to investigate if such processing is automatic or attention dependent.

Beat and temporal regularity perception have also been investigated with magnetoencephalography (MEG) and electroencephalography (EEG). For example, steady-state evoked potentials have been measured to capture the brains response to beat perception (Nozaradan et al., 2011, 2012). Moreover, several studies reported a correlation between neural oscillations and beat perception. Specifically, neural oscillations are correlated with the perception of beat (gamma frequency range: Snyder and Large, 2004), temporal regularity of a sound sequence (delta frequency range: Stefanics et al., 2010; Henry and Obleser, 2012), and the tempo of temporally regular sound sequences (beta frequency range: Fujioka et al., 2012). Fujioka et al. localized modulations in the beta frequency band with the tempo of temporally regular sounds to the auditory cortex, postcentral gyrus, left cingulate cortex, SMA, and left inferior temporal gyrus. Whether this indicates a functional coupling in these brain areas during beat perception will need to be investigated in future studies. Furthermore, measures of neural oscillations in animal species that do not show spontaneous motor synchronization but might nevertheless show the ability to perceive beat (such as the European starling) could help elucidate which of these brain areas are sufficient for beat perception.

Tempo perception has been investigated with neuroimaging mostly in the context of beat perception. Not surprisingly, these studies have associated tempo perception with some of the same brain regions implicated in temporal regularity perception, specifically the primary sensory and cortico-striatal brain areas (Thaut, 2003; Schwartze et al., 2011; Henry et al., 2013), and the neural oscillations therein (Fujioka et al., 2012). Beat and tempo processing may therefore rely on a similar neural network, which may or may not be partially distinct from that involved in temporal grouping perception. Studying tempo perception by measuring the average perceived rate of a temporally non-regular stimulus could reveal the underlying mechanism of tempo perception without the confounding influence of beat perception.

Neuroimaging studies investigating temporal grouping perception are yet sparse. Tasks referred to as rhythm discrimination have relied on the perception of temporal grouping and revealed the general network of timing related brain areas including the STG, (pre-)SMA, basal ganglia, and cerebellum (Grahn and Brett, 2007). There are several behavioral studies in humans that report behavioral measures for temporal grouping (Trehub and Thorpe, 1989; ten Hoopen et al., 2006; Geiser and Gabrieli, 2013), but, to our knowledge, these measures have not yet been implemented in neuroimaging studies. In the future, studies should elucidate whether specific regions of the cortico-striatal network play a role in temporal grouping perception and whether these are functionally distinguishable from the brain areas involved in beat and tempo perception.

## What are the potential neural mechanisms for encoding global timing information?

Before global timing information can be processed by multiple higher-order brain structures, the acoustic signal must be encoded in auditory cortex. In cats, inactivation of the anterior, but not the posterior, field of secondary auditory cortex impairs performance on a temporal pattern-matching task (Lomber and Malhotra, 2008) suggesting that the initial stage of temporal pattern processing may take place in a subset of higher auditory cortical fields. A neuron within auditory cortex can exhibit time-locked responses to repeated events (e.g., brief tones) as long as only one event falls within its temporal integration window. When multiple events fall within that window, that is, for faster occurring events, the firing rate of the neuron will co-vary with the number of events occurring within its temporal integration window (Lu et al., 2001; Anderson et al., 2006; Dong et al., 2011). This rate code represents the synthesis of multiple acoustic events and could, thus, reflect a first neural encoding mechanism toward computing global timing information.

In general, the temporal integration window in the auditory pathway varies. Previous reports suggest an integration windows of less than 10 ms in subcortical structures, ~20–25 ms in primary auditory cortex, and more than 100 ms in auditory fields downstream from primary auditory cortex in primates (Lu et al., 2001; Bartlett and Wang, 2007; Bendor and Wang, 2007; Walker et al., 2008). In human auditory cortex, two different processing timescales have been postulated: 25–50 and 200–300 ms (Boemio et al., 2005). Unlike other acoustic information related to music, such as pitch (Bendor and Wang, 2005; Bizley et al., 2013) and timbre (Walker et al., 2011), global timing requires the integration of temporal patterns spanning several seconds, thus exceeding the temporal integration window in primary or secondary auditory cortex of humans and non-human species. How does our brain solve this problem, and encode temporal patterns extending beyond a neuron's temporal integration window? One possibility is that these computations happen outside of auditory cortex in neurons with longer temporal integration windows (e.g., neurons sensitive to particular temporal patterns). However, evidence of such neurons is yet lacking. Alternatively, the time-locked neural activity to each acoustic event could first provide a passive representation of the global temporal pattern. Then, a distributed neuronal ensemble could perform higher-level timing computations on this temporal representation. While such timing computations have not been reported in auditory cortex, neural circuits capable of ensemble-based encoding of temporal or sequential patterns have been reported in two brain regions: (1) area HVC in song birds, and (2) the hippocampus of rodents. We speculate that similar mechanisms could be used to process the temporal patterns required for encoding global timing information and discuss these neural mechanisms in more detail below.

In the zebra finch, area HVC contains neurons that fire during the bird's song production in a mechanism referred to as *synfire* chain. Neural activity in a *synfire* chain is sparsely present throughout the song sequence, with each neuron being active at only one single time-point within the sequence and not necessarily correlated with the onset of a syllable (Hahnloser et al., 2002). At a population level, the spiking of neurons defines the tempo for the song's production. If HVC is cooled bilaterally, the interval between each sparse burst lengthens, effectively slowing down the neural representation of the song, and generating a slowed-down production of the bird's song (Long and Fee, 2008). Warming the HVC has the opposite effect. These data suggests that HVC acts as a clock for song timing. Intracellular recordings from HVC during song production indicate that the sequential pattern of neuronal firing is created by feed-forward excitation within a *synfire* chain (Long et al., 2010). Although HVC is only thought to play role in the production of a well-learned song (Aronov et al., 2008), one might speculate, that a neural mechanism similar to a *synfire* chain could be used for computing global timing information in other brain regions.

A different neural mechanism for encoding sequential information has been reported in the hippocampus of rodents. Neurons in the hippocampus are tuned to behaviorally relevant spatial (O'Keefe and Dostrovsky, 1971) and temporal information (Pastalkova et al., 2008; Kraus et al., 2013). A behavioral episode (e.g., a rat moving along a spatial trajectory) is encoded by the sequential activity of an ensemble of hippocampal neurons, with each neuron tuned to a different time point or location. This encoding interacts with the ongoing background theta (~8 Hz) oscillation in local field potentials by a process called “phase precession.” That is, the neuron fires a burst of spikes every theta cycle, with the timing of this burst gradually drifting earlier in each successive cycle. When the rodent enters a second neuron's place field, the relative phase of activity for the second neuron will consistently lag the firing of the first neuron. In each theta cycle, the two neurons consequently fire in a sequence matching the order the rodent passed through the two place fields. Since the time it takes to move through two overlapping place fields is longer than a theta cycle, phase precession effectively compresses a sequential pattern to fit the duration of a theta cycle (~125 ms). While the rodent is resting or sleeping, place cells spontaneously reactivate the temporally compressed version of this sequential pattern indicating that the hippocampus is capable of also storing the memory of a temporally compressed sequence (Lee and Wilson, 2002; Foster and Wilson, 2006). In principle, temporal compression of a sequence following the example of place field encoding and phase precession in the hippocampus could encode auditory temporal patterns spanning several seconds. Moreover, once a sequence is temporally compressed, neurons with shorter temporal integration windows (e.g., neurons in auditory cortex) could represent global timing information using a rate code. Recent evidence suggests that acoustical processing can influence hippocampal responses (Bendor and Wilson, 2012; Itskov et al., 2012). However, whether the hippocampus can encode acoustic temporal patterns in the same way it encodes space and time remains to be investigated.

## Conclusions

We suggest global timing as a conceptual framework to investigate the temporal aspects of musical rhythm perception that require listeners to integrate temporal patterns spanning several seconds. Music perception in humans comprises three features of global timing—temporal grouping, beat, and tempo,—which are perceptually distinguishable yet interdependent. Both humans and non-human species make use of temporal grouping and tempo processing in auditory signals. These features are ecologically important in interpreting the vocal calls of many species. In contrast, beat perception—that aspect of global timing that often distinguishes music from other sounds—appears to be limited in most non-human species. Global timing perception relies on neural processes across a wide network of subcortical and cortical brain regions, including the basal ganglia, SMA, and prefrontal cortex, as well as a subset of fields within auditory cortex. The neural mechanisms that underlie global timing perception remain elusive. However, given the limited temporal integration window of neurons, it is likely that sequential activity within a neuronal ensemble is required to encode global timing. Further research into the neural basis of processing outside of auditory cortex may help in understanding how the brain processes global timing. The examination of vocal learners such as parrots or European starlings, that could be capable of beat perception, could be essential toward that goal. In future experiments, designs that allow dissociating temporal grouping, beat and tempo perception processes will be a necessary step toward unraveling neural mechanisms of global timing.

### Conflict of interest statement

The authors declare that the research was conducted in the absence of any commercial or financial relationships that could be construed as a potential conflict of interest.
